# Ocular emotion discrimination disorders in self‐limited epilepsy patients with centrotemporal spikes complicated with electrical status epilepticus during sleep: A pediatric neuropsychological study

**DOI:** 10.1002/brb3.3056

**Published:** 2023-05-16

**Authors:** Chunmei Yang, Nan Jiang, Lulu Wu, Kaili Zhang, Xiaocui Wang, Bin Yang

**Affiliations:** ^1^ Department of Pediatric Neurology Anhui Provincial Children's Hospital Affiliated to Anhui Medical University, The Fifth Clinical College Affiliated to Anhui Medical University Hefei China

**Keywords:** electrical status epilepticus during sleep, eye area emotion recognition, self‐limited epilepsy with centrotemporal spikes

## Abstract

**Objective:**

We investigated the characteristics and factors influencing eye emotion recognition in self‐limited epilepsy patients with centrotemporal spikes (SeLECTS) complicated with electrical status epilepticus during sleep (ESES).

**Methods:**

We selected SeLECTS (*n* = 160) patients treated in the outpatient and inpatient departments of Anhui Children's Hospital from September 2020 to January 2022. According to the video electroencephalogram monitoring slow‐wave index (SWI), SeLECTS patients with SWI < 50% were assigned into the typical SeLECTS group (*n* = 79), and patients with SWI ≥ 50% were assigned into the ESES group (*n* = 81). Patients in the two groups were assessed by The Eye Basic Emotion Discrimination Task (EBEDT) and The Eye Complex Emotion Discrimination Task (ECEDT), respectively. Comparisons were made with age‐, sex‐ and education level‐matched healthy control participants. The correlation between the characteristics of emotional discrimination disorder in the eye area and the clinical influencing factors was analyzed in ESES group, and *p* ≤ .050 was the threshold for significance.

**Results:**

Relative to the healthy control group, scores of sadness and fear in the typical SeLECTS group were markedly lower (*p* = .018, *p* = .023), while differences in scores of disgust, happiness, surprise, and anger were not significantly different between the groups (*p* = .072, *p* = .162, *p* = .395, *p* = .380, respectively). Compared with the healthy control group, the ESES group had significantly low scores in recognition of sadness, fear, disgust, and surprise (*p* = .006, *p* = .016, *p* = .043 and *p* = .038, respectively). However, differences in recognition of happiness and anger between the groups were not significant (*p* = .665 and *p* = .272). Univariate logistic analysis showed that the score of eye recognition for sadness in the ESES group was affected by age of onset, SWI, ESES duration and number of seizures. The score of eye recognition for fear was mainly affected by SWI, while the score of eye recognition for disgust was affected by SWI and number of seizures. The surprised eye emotion recognition score was mainly affected by the number of seizures. Variables with *p* < .1 were considered to be independent variables of multivariable ordered logistic regression. Multivariate logistic analysis showed that sadness emotion recognition was mainly affected by SWI and ESES duration, while disgust was mainly affected by SWI.

**Conclusion:**

The typical SeLECTS group showed impaired emotion (sadness and fear) recognition function in the eye area. The ESES group was associated with more intense emotional (sadness, fear, disgust, and surprise) recognition impairment in the eye region. The higher the SWI, the younger the onset age and the longer the duration of ESES, while the more the number of seizures, the more serious the impairment of emotional recognition function in the affected eye area.

## Graphical abstract

SeLECTS complicated with ESES was associated with more extense emotional (sadness, fear, disgust, and surprise) recognition impairment in the eye region. The higher the SWI, the younger the onset age and the longer the duration of ESES, while the more the number of seizures, the more serious the impairment of emotional recognition function in the affected eye area.

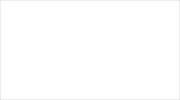

## 1 INTRODUCTION

Self‐limited epilepsy with centrotemporal spikes (SeLECTS) is the most common childhood idiopathic focal epilepsy syndrome, with a prevalence of about 10/210,000. It accounts for approximately 15%−24% of all epilepsy cases among children. The age of onset is majorly around 3–13 years old, the peak age of onset is around 5–8 years old, while spontaneous remission is about 2–4 years after onset (Lee et al., 2017). Electrical status epilepticus in sleep (ESES) is a special electroencephalogram (EEG) phenomenon, which refers to the continuous or nearly continuous epileptiform discharges of 1.5–2.5 Hz spike and slow waves during non‐rapid eye movement sleep (Yan & Xiaoming, 2018). This abnormal EEG phenomenon was first proposed by Patry et al. in 1971, and named as electrical status epileptiform during sleep in children by Tassinari et al. (1992). Its hallmark features are age dependence and self‐limitation. In clinical practice, ESES patients manifest diverse symptoms such as, neuropsychological impairment, including language, behavior, learning, memory, attention, social interaction, motor skills, and cognitive. Even if EEG paradoxical discharge and seizures are controlled in children with ESES, neurocognitive and behavioral deterioration may be permanent (Loddenkemper et al., 2011).

Spike‐wave index (SWI) in sleep stage is an EEG index for describing the severity of ESES, which refers to the percentage of the duration of all abnormal spines during the first non‐rapid eye movement (NREM) stage (Tarokh et al., 2014). It can utilized as part of the diagnostic criteria of ESES, but the corresponding quantification criteria are not given in all of the current studies. Tassinari et al. (2009) suggested that SWI of ESES should reach 85%−100%, and most clinicians use SWI ≥ 50% as the diagnostic criteria for ESES. The ESES defined in this article are SWI ≥ 50%. Eye area emotion recognition is used to infer the inner view, purpose and attitude of others via various clues in the eye area, which plays an important role in development of social emotions among children. However, it has not been established whether SeLECTS patients develop ocular emotional discrimination disorders during seizures, and whether SeLECTS patients with ESES (SWI ≥ 50%) have more severe ocular emotional discrimination disorders than SeLECTS patients (SW < 50%). Therefore, we conducted a study on emotional discrimination in eye regions of patients with SeLECTS complicated with ESES to establish the clinical factors and characteristics of potential emotional recognition disorders.

## 2 SELECTION OF PARTICIPANTS AND METHODS

### 2.1 Study participants

A total of 79 children (46 males and 33 females, with a median age of 8 (7, 9) years) diagnosed with the typical SeLECTS (SWI < 50%) at Anhui Provincial Children's Hospital from September 2020 to January 2022 were enrolled in this study. A total of 81 patients (44 males and 37 females, with a median age of 8 (7, 9) years) diagnosed with SeLECTS combined with ESES (SWI ≥ 50%) (ESES group) in our hospital during the same period were also enrolled. A total of 71 healthy children (49 boys and 22 girls, median age 8 (7, 9) years) who underwent physical examination in our hospital and found not to have hearing disorders or mental or nervous system diseases were enrolled as the healthy control group. Differences in gender, age, and years of education among the three groups were not significant (*p* > .050).

### 2.2 Inclusion criteria for the typical SeLECTS group

The inclusion criteria for this group were as follows: (i) patients who met the SeLECTS diagnostic criteria per the International Anti‐Epileptic Alliance (ILAE) in 2022; (ii) the spike‐wave and spike‐slow wave in the Rolandic area were detected by 24‐h VEEG, and the SWI in the first NREM period was less than 50%; (iii) patients who had no history of asphyxia at birth or a previous history of mental disorders or congenital diseases; (iv) patients who had no abnormalities in vision, hearing and color recognition.

### 2.3 Inclusion criteria for the ESES group

The inclusion criteria for this group were as follows: (i) patients who met the SeLECTS diagnostic criteria per the International Anti‐Epileptic Alliance (ILAE) in 2022; (ii) the 24‐h long range video EEG monitoring method, which included a complete sleep physiological cycle, was used to detect continuous or nearly continuous emission of the spikes and slow spikes in the Rolandic area during sleep, and the SWI reached or exceeded 50% in the first NREM phase; (iii) patients who had no history of birth injury, asphyxia or a previous history of mental illness and congenital diseases; and (iv) patients who had no abnormalities in vision, hearing, and color recognition.

### 2.4 Inclusion criteria for the healthy control group

The inclusion criteria for the healthy control group were as follows: (i) Individuals whose Minimum Mental State Examination (MMSE) scores were between 17 and 30 points and (ii) Individuals who had no family history of epilepsy or a history of convulsions.

### 2.5 Exclusion criteria

The exclusion criteria for this study were as follows: (i) Individuals whose scores of MMSE were less than 17 points; (ii) there were intracranial space occupying lesions or lesions in the cranial imaging examination; (iii) patients with acute or other chronic diseases and systemic organic diseases; and (iv) patients with Landau‐Kleffner syndrome(LKS), infantile spasm, epilepsy with continuous spike and wave during slow‐wave sleep(CSWS) and other syndromes that may have been accompanied by ESES.

## 3 METHODS

### 3.1 Clinical data collection

We collected relevant clinical data by questionnaire. We recorded the age, sex, age of first seizure, education level, family history of related neuropsychiatric diseases, number of seizures, duration of ESES, performance of seizures, duration of each seizure, number of febrile seizures, EEG discharge site, SWI, cognitive and other clinical data for all patients at the time of enrollment in this study; moreover, we also collected some clinical auxiliary examination data, including imaging findings available by clinical charts recorded, such as cranial magnetic resonance imaging(MRI). In addition, clinical data such as electrocardiogram, biochemistry and blood routine were recorded by clinical charts.

### 3.2 Scoring criteria for The Eye Basic Emotion Discrimination Task

**FIGURE 1:**
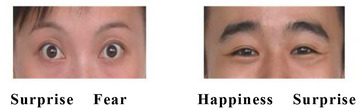
Pictures of The Eye Basic Emotion Discrimination Task.

Eye emotion discrimination analyses for the ESES group, the typical SeLECTS group and the healthy control participants were respectively performed using The Eye Basic Emotion Discrimination Task (EBEDT) (Adolphs et al., 2002). The test involves 120 eye expressions, including six basic eye expressions: anger, happiness, fear, sadness, disgust, and surprise. During the test, pictures were presented in random form on a computer, each containing an image of the eye, and two basic emotional words (Figure 1). Participants were asked to select the emotion they thought the person in the picture was experiencing, and a correct choice was 1 point. The highest score of each eye area emotion was 20 points, and the total score of the six basic emotions was 120 points. The number of correct choices for each emotion and the total number of correct emotions were calculated separately.

### 3.3 Scoring criteria for The Eye Complex Emotion Discrimination Task

**FIGURE 2:**
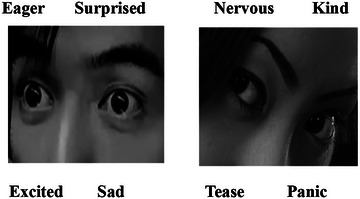
Pictures of The Eye Complex Emotion Discrimination Task.

Eye emotion discrimination analyses for the ESES group, typical SeLECTS group and healthy control participants were respectively performed and analyzed using The Eye Complex Emotion Discrimination Task (ECEDT) (Adolphs et al., 2002). This test involved 34 black and white photos of Chinese faces (17 men and 17 women), showing emotions such as worry, sadness, satisfaction, and expectation. Participants were asked to select the emotion they thought the person in the picture was experiencing, and to assess the gender of the subjects in the images (Figure 2). When both emotion and sex were correctly identified, the score was 1, with a potential maximum score of 34.

### 3.4 Statistical analysis

Statistical analyses were performed using the SPSS25.0 software. The normality for data distribution were tested by a histogram and Shapiro–Wilk test. Normally distributed data are expressed as mean ± standard deviation, while data that did not conform to normal distribution are expressed as medians or quartiles [*M* (Q25, Q75)]. The Mann–Whitney *U t*‐test was used to compare data between groups. Enumeration data are expressed as the number of cases. All variables were initially analyzed by univariate ordered logistic regression analysis, and variables with *p* < .1 were used as covariates in multivariate ordered logistic regression. Factors that were related to emotion recognition disorders were analyzed by multiple‐ordered logistic regression.

## 4 EXPERIMENTAL RESULTS

### 4.1 Characteristics of patients

#### 4.1.1 Clinical characteristics of patients in the typical SeLECTS group and the ESES group

The SeLECTS group consisted of a total of 79 children, including 46 males and 33 females, SWI less than 50% in the Rolandic area, with a median age of onset 6 (5, 8) years, a median age of enrollment 8 (7, 9) years, and a median of 3 (2, 4) years of education. The median number of seizures was 3 (3, 3.5), 17 cases were abnormal in the left Rolandic area, 22 cases were abnormal in the right Rolandic area, 40 cases were bilateral Rolandic area, 45 patients were focal seizures, and 34 patients were generalized seizures. Of these, 11 patients had a previous history of febrile convulsions, 4 patients had a family history of SeLECTS, and 6 patients had a family history of febrile convulsions. All patients had normal cranial imaging.

The ESES group consisted of 81 children, including 44 males and 37 females. Clinical data are summarized in Table 1. The median SWI value during NREM was 70% (60%, 80%), the median age of onset was 5 (4, 7) years, the median age at enrollment was 8 (7, 9) years, the median years of education was 3 (2, 4) years, and the median number of seizures was 5 (3, 11). The median duration of ESES was 12 (9.5, 16) months. There were 16 cases of abnormal discharge in the left Rolandic area, 19 cases in the right Rolandic area, and 46 cases in the bilateral Rolandic area. Among them, 20 patients had a previous history of febrile convulsions, 2 patients had a family history of SeLECTS, 8 patients had a family history of febrile convulsions, and 2 patients had a family history of psychiatric disorders. The cranial imaging of 81 patients was normal. Clinical data are summarized in Table 1.

**TABLE 1:** Clinical characteristics of patients in typical SeLECTS group and the ESES group.

Clinical data	Typical SeLECTS group (*N* = 79)	ESES group (*N* = 81)
Sex (male/female)^a^	46/33	44/37
Duration of each episode (minutes)^b^	1.5(1.3, 2.5)	2(0.8, 4.5)
Age of onset (years)^b^	6(5, 8)	5(4, 7)
Age at enrollment (years)^b^	8 (7, 9)	8 (7, 9)
Years of education (years)^b^	3 (2, 4)	3 (2, 4)
SWI (%)^b^	<50	70 (60, 80)
The number of seizures (times)^b^	3 (3, 3.5)	5 (3, 11)
ESES duration (months)^b^	–	12(9.5, 16)
Electroencephalogram^a^		
Left hemisphere spikes	17	16
Right hemisphere spikes	22	19
Bilateral hemisphere spikes	40	46
History of febrile seizures^a^	11	20
Seizure pattern^a^		
Focal seizure	45	49
Comprehensive seizure	34	32
Family history of neuropsychiatric diseases^a^		
Family history of epilepsy	4	2
Family history of febrile convulsion	6	8
Family history of psychiatric diseases	0	2

^a^ Counting data are represented by the number of cases.

^b^ Nonnormally distributed data are expressed as [*M* (Q25, Q75)].

### 4.2 Results of eye emotion discrimination task in patient groups

#### 4.2.1 Results of eye region discrimination task in typical SeLECTS group

**FIGURE 3:**
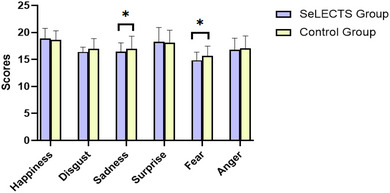
Comparison of basic eye area emotion recognition scores between the typical SeLECTS and healthy control groups.

Scores of patients in the typical SeLECTS group on recognition of sadness and fear were lower than those of the healthy control group (*p* = .018, *p* = .023). Differences between the two groups in the recognition scores of happiness, anger, disgust, and surprise were insignificant (*p* = .162, *p* = .380, *p* = .072, *p* = .395). Differences in EBEDT score and ECEDT between the SeLECTS group and the healthy control group were not significant (*p* = .061) and (*p* = .922) (Figure 3 and Table 2).

**TABLE 2:** Comparison of basic emotion recognition scores between the typical SeLECTS and control groups [*M* (Q25, Q75], *z*, *p*.

Group	Happiness	Disgust	Sadness	Surprise	Fear	Anger	EBEDT	ECEDT
SeLECTS group (*n* = 79)	20.0 (18.5, 20.0)	16.0 (16.0, 17.0)	17.0 (16.0, 17.5)	19.0 (18.0, 20.0)	15.0 (14.5, 16.0)	18.0 (16.0, 18.0)	102.0 (98.5, 105.0)	18.0 (15.5, 22.0)
Control group (*n* = 71)	19.0 (18.0, 20.0)	17.0 (16.0, 19.0)	18.0 (15.0, 19.0)	19.0 (18.0, 20.0)	16.0 (14.0, 17.0)	18.0 (16.0, 19.0)	105.0 (96.0, 110.0)	18.0 (14.0, 22.0)
*z*	−1.397	−1.799	−2.361	−0.851	−2.273	−0.879	−1.873	−0.097
*p*	.162	.072	.018*	.395	.023*	.380	.061	.922

* *p* ≤ .050 means the difference is statistically significant.

#### 4.2.2 Results of eye emotion discrimination task in ESES group

**FIGURE 4:**
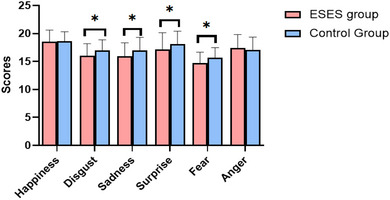
Comparison of basic eye area emotion recognition scores between the ESES and healthy control groups.

Scores of sadness, fear, disgust, and surprise in ESES group were significantly lower than those of the healthy control group (*p* = .006, *p* = .016, *p* = .043 and *p* = .038). Differences in scores of happiness and anger discrimination between the groups were not significant (*p* = .665, *p* = .272). The score for EBEDT in the ESES group was significantly lower than those of the healthy control group (*p* = .041). Differences in ECEDT between the two groups were insignificant (*p* = .697). These results are presented in Table 3 and Figure 4.

**TABLE 3:** Comparison of basic emotion recognition scores between the ESES and control groups [*M* (Q25, Q75], *z*, *p*.

Group	Happiness	Disgust	Sadness	Surprise	Fear	Anger	EBEDT	ECEDT
ESES group (*n* = 81)	20.0 (17.0, 20.0)	16.0 (14.0, 18.0)	16.0 (14.0, 18.0)	18.0 (16.0, 19.0)	15.0 (14.0, 16.0)	18.0 (16.0, 19.0)	102.0 (95.5, 107.0)	18.0 (16.0, 22.0)
Control group (*n* = 71)	19.0 (18.0, 20.0)	17.0 (16.0, 19.0)	18.0 (15.0, 19.0)	19.0 (18.0, 20.0)	16.0 (14.0, 17.0)	18.0 (16.0, 19.0)	105.0 (96.0, 110.0)	18.0 (14.0, 22.0)
*z*	−0.433	−2.029	−2.742	−2.072	−2.419	−1.097	−2.039	−0.389
*p*	.665	.043*	.006*	.038*	.016*	.272	.041	.697

* *p* ≤ .050 means the difference is statistically significant.

### 4.3 Clinical factors related to eye emotion discrimination for patients in the ESES group

Univariate analysis showed that age of onset (*p* = .025), duration of ESES (*p* = .004), SWI index (*p* = .003), and number of seizures (*p* = .042) were associated with sadness discrimination scores. Scores for fear recognition were correlated with the SWI index (*p* = .003), while scores for disgust recognition were correlated with the SWI index (*p* = .003) The number of seizures (*p* = .030). The score for surprise was associated with the number of seizures (*p* = .010) (Table 4). In univariate analysis, the factors found to be associated with eye emotion recognition and discrimination were selected as independent variables for multiple‐ordered regression, while scores for sadness, fear, disgust, and surprise were the dependent variables. Multiple‐ordered regression analysis showed that the longer the duration of ESES (*p* = .043), the higher the SWI index (*p* = .031) and the lower the sadness recognition score; the higher the SWI index (*p* = .014), the lower the disgust recognition score (Table 5).

**TABLE 4:** Univariate ordered logistic analysis results.

Variable	Sadness			Fear		
	OR	*p*	95%CI	OR	*p*	95%CI
Sex	1.346	.469	0.602–3.010	1.581	.275	0.694–3.602
Duration of each episode	0.982	.223	0.882–1.080	1.012	.585	0.969–1.058
Age of onset	1.250	.025*	1.028–1.520	0.894	.259	0.736–1.086
Age at enrollment	0.915	.425	0.735–1.139	1.039	.737	0.832–1.297
Years of education	1.159	.185	0.932–1.442	1.026	.820	0.498–4.087
SWI	0.847	.003*	0.814–0.881	0.558	.003*	0.250–0.768
The number of seizures	0.947	.042*	0.898–0.998	1.036	.168	0.985–1.162
ESES duration	0.868	.004*	0.788–0.957	1.061	.208	0.968–3.589
Electroencephalogram	0.715	.504	0.267–1.915	0.945	.914	0.343–2.606
History of febrile seizures	0.971	.951	0.384–2.458	2.240	.460	0.273–3.589
Seizure pattern	1.998	.102	0.872–4.576	0.751	.502	0.325–1.736
Family history of neuropsychiatric diseases	0.787	.642	0.287–2.162	1.426	.509	1.398–3.589

Variable	Disgust			Surprise		
	OR	*p*	95%CI	OR	*p*	95%CI
Sex	1.536	.311	0.670–3.524	1.036	.944	0.383–2.804
Duration of each episode	0.999	.957	0.955–1.045	0.408	.143	0.123–1.356
Age of onset	0.961	.688	0.792–1.166	0.947	.686	0.726–1.235
Age at enrollment	1.065	.580	0.852–1.332	1.295	.860	0.074–2.648
Years of education	1.067	.565	0.856–1.329	0.927	.958	0.055–1.639
SWI	0.058	.003*	0.015–0.987	0.995	.823	0.955–1.038
The number of seizures	0.060	.030*	0.016–0.117	0.388	.010*	0.320–0.560
ESES duration	0.940	.189	0.857–1.031	0.956	.402	0.860–1.062
Electroencephalogram	1.297	.611	0.476–3.537	1.404	.558	0.451–4.370
History of febrile seizures	0.709	.476	0.275–1.828	1.534	.483	1.464–5.073
Seizure pattern	0.541	.162	0.229–1.279	0.465	.112	0.180–1.196
Family history of neuropsychiatric diseases	1.309	.620	0.452–3.786	0.531	.222	0.192–1.468

* *p* ≤ .050 means the difference is statistically significant.

**TABLE 5:** Multiple‐ordered logistic analysis results.

Independent variables	Independent variables	OR	*p*	95%CI
Sadness	Age of onset	1.120	.295	0.906–1.383
	ESES duration	0.899	.043*	0.811–0.997
	SWI	0.859	.031*	0.829–0.933
	The number of seizures	0.976	.409	0.923–1.033
Disgust	SWI	0.850	.014*	0.810–0.892
	The number of seizures	0.206	1.036	0.181–1.094

* *p* ≤ .050 means the difference is statistically significant.

## 5 DISCUSSION

Sleep activation of epileptiform discharges is very common in SeLECTS. In SeLECTS children, learning and behavioral disorders may also occur, but generally, there is no serious intellectual regression (Vinayan et al., 2005). Currently, it has not been conclusively determined whether some SeLECTS can evolve into ESES (Uliel‐Sibony & Kramer, 2015). The diagnosis of ESES should be considered if abnormal spikes or an increase of slow spikes during sleep and evolution of cognitive and/or behavioral degeneration occur in SeLECTS patients during the disease course. Therefore, sufficient attention and awareness should be given to patients with SeLECTS complicated by ESES.

Eye area emotion recognition is a method for assessing other people's thoughts, intentions, emotions, and other abilities. Impaired emotional recognition abilities may lead to abnormalities in social cognition and behaviors, resulting in a negative impact on social life. Theory of mind (ToM) is a higher cognitive process, which is one of the core contents of social cognition. The damage of ToM may negatively affect the social life and interpersonal communication. Stewart et al. (2016) believed that early theory of mind damage may have a cumulative negative impact on social skills that continued to develop in adulthood. Even if the seizure stopped and EEG was completely alleviated, cognitive damage often persists. In their studyCassel et al. (2019) found that in mental diseases, situational, collaborative and empirical interventions can improve patients' social cognition to a large extent. By assessing the characteristics and related clinical factors of ocular emotion recognition disorders in patients with SeLECTS and ESES, we found that patients with SeLECTS and ESES had impaired ocular emotion recognition abilities; thus, it is important to provide them with situational, cooperative, empirical repetitive stimulation, and other early intervention measures.

Wu et al. (2021) found that SeLECTS patients may show cognitive barriers to sadness, fear, and disgust. Our results demonstrated that, compared with the same control group, the typical SeLECTS group had a special eye area emotion discrimination disorder, which was mainly related to sadness and fear, but there were no significant differences in recognition of happiness, disgust, surprise, and anger. Emotional differences in emotion recognition in the eyes of ESES patients is not extensive but is associated with specific emotion recognition of sadness, fear, disgust, and surprise. Differences in emotion recognition of joy and anger between patients with ESES and healthy controls were insignificant. Consistent with our findings, it has been reported that the most common cognitive impairment of focal epilepsy is cognitive impairment of fear, followed by cognitive impairment of sadness and disgust, while cognitive impairment of anger and surprise is relatively small (Monti & Meletti, 2015). The amygdala plays an important role in facial expression recognition of emotional content (Gordon, 2000). The amygdala and ventromedial prefrontal cortex exert strong bidirectional projection functions via the uncinate fasciculus muscle. These areas are two important nodes in emotion recognition network. During emotion recognition, the amygdala plays an important role in guiding people to focus on facial emotion prominent areas, especially the eyes, which play an important role in controlling visual attention, while the thalamus is associated with impairment of negative emotion recognition. Liza (2022) found that mSTN‐CRH neurons are the key pivot of the common loop regulating fear and sleep. The corticotropin releasing hormone in the medial subthalamic nucleus is produced by neurons under the control of acute fear responses, and it mediates the REM sleep state caused by continuous pressure. Xiaoting et al. (2018) found that the thalamus is involved in emotion recognition and regulates emotional memory processing. The emotional memory of thalamic patients is impaired, mainly in terms of neutral and negative emotion recognition. With advances in neuroimaging, it has been confirmed that children with SeLECTS have abnormal developments in the frontal, temporal, amygdala, hypothalamus, and other brain regions. Sanchez et al. (2017) found that the relative thalamus volume of ESES patients with normal MRI was smaller and was associated with age and total brain volume. After correcting for age and brain volume, the difference in right thalamus reached statistical significance, while that of the left thalamus exhibited a marginal significance. In animal models, the interruption of neural transmission in the corticothalamic cortical spindle generating circuit led to generalized spike waves, which is comparable to the findings in ESES. Early ESES onset among patients may be attributed to the thalamic neurotransmission disorder (Beenhakker & Huguenard, 2009), which is associated with the disorder of SeLECTS patients with ESES in recognizing sadness, fear, aversion, and surprise.

Our logistic analysis showed that the higher the SWI, the lower the scores for sadness, fear, and disgust recognition. Recently, Huber et al. (2004) reported that an increase in local slow‐wave activity (SWA) in sleep after learning results in improvement of learning tasks. Based on these findings, we postulated that ESES interferes with local SWA of the epileptic focus, impairs the neural transmission process, and may damage the local cognitive functions that are related to learning and other processes. In their study on SeleCTS, Massa et al. (2001) showed that the disorder of higher cortical functions is related to the continuous existence of a large number of spikes during sleep. We also found that the age of patients was negatively correlated with scores of sad emotion recognition, and the younger the age of onset, the more impaired the sad emotion recognition ability. The recognition ability of negative emotions is seriously damaged in the early stages of epileptic seizures, and the younger the age of onset, the more serious the damage to negative emotion recognition. Ciumas et al. (2017) postulated that delayed development of neural networks may affect the cognitive functions of patients.

Bora and Meletti (2016) found that epileptic patients have obvious defects in the theory of mind and facial emotion recognition. Earlier onset was associated with mental theory impairment, and the right‐side attack was mainly associated with cognitive impairment of fear, sadness, and disgust. Meletti et al. (2003) found that the severity of emotion recognition disorders was associated with age at the time of first seizure, and the right temporal lobe discharge of early seizure was a key factor in the serious defect of fear recognition. However, in our study, we found no significant correlation between epileptic discharge areas and eye emotion recognition scores.

High SWI and long‐term ESES are the main predictors of poor prognosis of epilepsy (Veggiotti et al., 2002). Scholtes et al. (2005) reported that cognitive decline is associated with long‐term ESES and early epileptic activities. We found that the longer the patient's ESES lasted, the more severe the impairment of sadness emotion recognition. Therefore, we believe that the duration of ESES may be associated with cognitive and behavioral deterioration. The longer the duration of ESES, the severer the impairment of eye area emotion recognition abilities.

In addition, we found that the number of seizures is associated with specific emotional disorders. The more seizures, the more significant the impairment of sadness, disgust, and surprise emotion recognition abilities, consistent with findings from previous studies. Fuerst et al. (2003) identified a negative correlation between sad emotion recognition scores and the number of seizures, with patients with more epileptic seizures having more severely impaired synaptic connections between brain microstructure and adjacent neurons, which may lead to more frequent seizures and more impaired sad emotion recognition. According to the research conducted by van den Munckhof et al. (2020), there was a significant impairment in the intelligence, memory, and executive abilities of children who experienced a higher frequency of seizures. Through various mental theory assessments of 79 epileptic patients, including 45 cases of focal epilepsy and 34 cases of generalized epilepsy, Morou et al. (2018) found that compared with 70 normal healthy people, all mental theories were significantly impaired in patients with focal epilepsy, while in generalized epilepsy, there were only minor abnormalities in individual tasks. Thus, focal epilepsy is closely correlated with abnormalities of the whole mental theory ability. However, in this study, we did not find a significant relationship between seizure patterns and ocular emotion recognition scores.

In this study, ECEDT scores for patients in the affected group were not significantly different from those of healthy controls, which may be due to the younger age of the affected group and control group, the incomplete maturity of the complex emotion recognition system in the eye region, and the inability of both groups to accurately identify complex emotions.

In summary, higher SWI and longer ESES duration are the main factors associated with impairment of eye area emotion recognition functions. Oztoprak et al. (2021) showed that improvement in SWI was associated with better cognition, and most patients' cognitive functions improved after SWI improvement. Therefore, even though in most cases, epilepsy subsides over time in patients with SeLECTS complicated with ESES, many children still have serious cognitive, language, and theory of mind impairments. Early detection and effective treatment are necessary to improve the long‐term prognosis of the disease. Second, with advances in imaging, neuropsychology and other disciplines, different neuropsychological experiments can be used to evaluate the neuropsychological characteristics of various diseases, to progressively understand the neurocognitive functions and social adaptive functions of different patients. The characteristics of cognitive impairment can be used to identify the brain regions with possible functional abnormalities. In this study, in the eye area for basic emotion recognition in children with SeLECTS and ESES, there were specific abnormalities in the recognition of fear, sadness, disgust, and shock eye emotions, which may be attributed to abnormalities in the thalamus, amygdala, and other brain regions. Therefore, brain structure and functional abnormalities in children may be the common pathogenetic factors for emotional damage in the eyes of SeLECTS and ESES patients. In addition, this study has some limitations. First, we used a relatively small sample size. Second, although we studied the influence of the age of first onset of epilepsy, the number of seizures and abnormal discharge index, the location of abnormal discharge, and the form of seizures on emotional recognition, the effects of antiepileptic drugs on emotional recognition were not determined. Third, we did not conduct a detailed assessment of variant SeLECTS. Patients with variant SeLECTS exhibited severer cognitive impairment, which may affect their emotion recognition. Finally, it is known that emotions are not universal and are context‐dependent: A person can cry when experiencing sadness, but they can also cry for joy. Therefore, showing a person an image of someone crying does not invariably mean that the person in the image is sad. According to Adolphs et al. (2019), the perceiver's brain makes sense of data meaningful by categorizing it, and in doing so imposes a socially agreed‐upon emotional function. In a given moment, my brain might categorize my sense data as an instance of sadness and your brain might categorize the sense data coming from me—my movements, my vocal acoustics, in the same context—as an instance of anger. We can compare our inferences to one another, or what is normative in that particular situation, but there are no objective criteria to say who is right. In addition, this study is completely psychological and behavioral. Future studies should assess the mechanisms of emotion recognition disorders by combining neuroimaging and electrophysiological technologies.

## 6 CONCLUSION

In the typical SeLECTS group, eye area emotion recognition function was impaired, mainly sadness and fear emotion recognitions. The emotion recognition function of eyes for patients with SeLECTS complicated with ESES was severely damaged, particularly in terms of emotional recognition of sadness, fear, disgust, and surprise. The higher the SWI, the younger the age of onset, the longer the duration of ESES, and higher the number of attacks, the severer is the impairment of eye area emotion recognition function.

## AUTHOR CONTRIBUTIONS

Bin Yang designed the study. Chunmei Yang wrote the manuscript. Kaili Zhang and Xiaocui Wang analyzed the EEG data. Lulu Wu, Nan Jiang revised the manuscript and directed the research. All authors agreed to the publication of the final version of the manuscript.

## FUNDING

This research did not receive any specific grant from funding agencies in public, commercial, or not‐for‐profit sectors.

## CONFLICT OF INTEREST STATEMENT

The author declares that there is no potential conflict of interest with the research, authorship, and publication of this article.

### PEER REVIEW

The peer review history for this article is available at https://publons.com/publon/10.1002/brb3.3056.

## Data Availability

The data sets generated during and/or analyzed during the current study are not publicly available, but are available from the corresponding author on reasonable request.

## References

[brb33056-bib-0001] Adolphs, R. , Baron‐Cohen, S. , & Tranel, D. (2002). Impaired recognition of social emotions following amygdala damage. Journal of Cognitive Neuroscience, 14(8), 1264–1274. 10.1162/089892902760807258 12495531

[brb33056-bib-0002] Adolphs, R. , Mlodinow, L. , & Barrett, L. F. (2019). What is an emotion? Current Biology, 29(20), R1060–R1064. 10.1016/j.cub.2019.09.008 31639344 PMC7749626

[brb33056-bib-0003] Beenhakker, M. P. , & Huguenard, J. R. (2009). Neurons that fire together also conspire together: Is normal sleep circuitry hijacked to generate epilepsy? Neuron, 62(5), 612–632. 10.1016/j.neuron.2009.05.015 19524522 PMC2748990

[brb33056-bib-0004] Bora, E. , & Meletti, S. (2016). Social cognition in temporal lobe epilepsy: A systematic review and meta‐analysis. Epilepsy & Behavior, 60, 50–57. 10.1016/j.yebeh.2016.04.024 27179192

[brb33056-bib-0005] Cassel, A. , Mcdonald, S. , Kelly, M. , & Togher, L. (2019). Learning from the minds of others: A review of social cognition treatments and their relevance to traumatic brain injury. Neuropsychological Rehabilitation, 29(1), 22–55. 10.1080/09602011.2016.1257435 27899036

[brb33056-bib-0006] Ciumas, C. , Laurent, A. , Saignavongs, M. , Ilski, F. , De Bellescize, J. , Panagiotakaki, E. , Ostrowsky‐Coste, K. , Arzimanoglou, A. , Herbillon, V. , Ibarrola, D. , & Ryvlin, P. (2017). Behavioral and fMRI responses to fearful faces are altered in benign childhood epilepsy with centrotemporal spikes (BCECTS). Epilepsia, 58(10), 1716–1727. 10.1111/epi.13858 28762475

[brb33056-bib-0007] Fuerst, D. , Shah, J. , Shah, A. , & Watson, C. (2003). Hippocampal sclerosis is a progressive disorder: A longitudinal volumetric MRI study. Annals of Neurology, 53(3), 413–416. 10.1002/ana.10509 12601713

[brb33056-bib-0008] Gordon, N. (2000). Cognitive functions and epileptic activity. Seizure: The Journal of the British Epilepsy Association, 9(3), 184–188. 10.1053/seiz.2000.0390 10775514

[brb33056-bib-0009] Huber, R. , Felice Ghilardi, M. , Massimini, M. , & Tononi, G. (2004). Local sleep and learning. Nature, 430(6995), 78–81. 10.1038/nature02663 15184907

[brb33056-bib-0010] Lee, Y. J. , Hwang, S. U. K. , & Kwon, S. (2017). The clinical spectrum of benign epilepsy with centro‐temporal spikes: A challenge in categorization and predictability. Journal of Epilepsy Research, 7(1), 1–6. 10.14581/jer.17001 28775948 PMC5540684

[brb33056-bib-0011] Liza, L. (2022). Study on the neural circuit of subthalamic nucleus regulating fear and sleep. University of Chinese Academy of Sciences Shenzhen Institute of Advanced Technology, Chinese Academy of Sciences.

[brb33056-bib-0012] Loddenkemper, T. , Fernández, I. S. , & Peters, J. M. (2011). Continuous spike and waves during sleep and electrical status epilepticus in sleep. Journal of Clinical Neurophysiology, 28(2), 154–164. 10.1097/WNP.0b013e31821213eb 21399511

[brb33056-bib-0013] Massa, R. , De Saint‐Martin, A. , Carcangiu, R. , Rudolf, G. , Seegmuller, C. , Kleitz, C. , Metz‐Lutz, M. N. , Hirsch, E. , & Marescaux, C. (2001). EEG criteria predictive of complicated evolution in idiopathic rolandic epilepsy. Neurology, 57(6), 1071–1079. 10.1212/WNL.57.6.1071 11571336

[brb33056-bib-0014] Meletti, S. , Benuzzi, F. , Rubboli, G. , Cantalupo, G. , Stanzani Maserati, M. , Nichelli, P. , & Tassinari, C. A. (2003). Impaired facial emotion recognition in early‐onset right mesial temporal lobe epilepsy. Neurology, 60(3), 426–431. 10.1212/WNL.60.3.426 12578923

[brb33056-bib-0015] Monti, G. , & Meletti, S. (2015). Emotion recognition in temporal lobe epilepsy: A systematic review. Neuroscience and Biobehavioral Reviews, 55, 280–293. 10.1016/j.neubiorev.2015.05.009 25999121

[brb33056-bib-0016] Morou, N. , Papaliagkas, V. , Markouli, E. , Karagianni, M. , Nazlidou, E. , Spilioti, M. , Afrantou, T. , Kimiskidis, V. K. , Foroglou, N. , & Kosmidis, M. H. (2018). Theory of Mind impairment in focal versus generalized epilepsy. Epilepsy & Behavior, 88, 244–250. 10.1016/j.yebeh.2018.09.026 30317058

[brb33056-bib-0017] Öztoprak, Ü. , Yayici Köken, Ö. , Aksoy, E. , & Yüksel, D. (2021). Spike‐wave index assessment and electro‐clinical correlation in patients with encephalopathy associated with epileptic state during slow sleep (ESES /CSWS); single‐center experience. Epilepsy Research, 170, 106549. 10.1016/j.eplepsyres.2021.106549 33450525

[brb33056-bib-0032] Patry, G. , Lyagoubi, S. , Tassinari, C. A. , & Yüksel, D. (1971). Subclinical "electrical status epilepticus" induced by sleep in children. A clinical and electroencephalographic study of six cases[J]. Arch Neurol, 24(3), 242–252. 5101616 10.1001/archneur.1971.00480330070006

[brb33056-bib-0018] Sánchez Fernández, I. , Peters, J. M. , Akhondi‐Asl, A. , Klehm, J. , Warfield, S. K. , & Loddenkemper, T. (2017). Reduced thalamic volume in patients with Electrical Status Epilepticus in Sleep. Epilepsy Research, 130, 74–80. 10.1016/j.eplepsyres.2017.01.010 28160673

[brb33056-bib-0019] Scholtes, F. B. J. , Hendriks, M. P. H. , & Renier, W. O. (2005). Cognitive deterioration and electrical status epilepticus during slow sleep. Epilepsy & Behavior, 6(2), 167–173. 10.1016/j.yebeh.2004.11.001 15710299

[brb33056-bib-0020] Stewart, E. , Catroppa, C. , & Lah, S. (2016). Theory of mind in patients with epilepsy: A systematic review and meta‐analysis. Neuropsychology Review, 26(1), 3–24. 10.1007/s11065-015-9313-x 26797753

[brb33056-bib-0021] Tarokh, L. , Carskadon, M. A. , & Achermann, P. (2014). Early adolescent cognitive gainsare marked by increased sleep EEG coherence. PLoS One, 9(9), e106847. 10.1371/journal.pone.0106847 25208326 PMC4160237

[brb33056-bib-0022] Tassinari, C. A. , Cantalupo, G. , Rios‐Pohl, L. , Giustina, E. D. , & Rubboli, G. (2009). Encephalopathy with status epilepticus during slow sleep: “the Penelope syndrome”. Epilepsia, 50(7), 4–8. 10.1111/j.1528-1167.2009.02209.x 19682041

[brb33056-bib-0023] Tassinari, C. A. , Michelucci, R. , Forti, A. , Salvi, F. , Plasmati, R. , Rubboli, G. , Bureau, M. , Dalla Bernardina, B. , & Roger, J. (1992). The electrical status epilepticus syndrome. Epilepsy Research. Supplement, 6, 111–115. 1418468

[brb33056-bib-0024] Uliel‐Sibony, S. , & Kramer, U. (2015). Benign childhood epilepsy with Centro‐Temporal spikes (BCECTSs), electrical status epilepticus in sleep (ESES), and academic decline–how aggressive should we be? Epilepsy & Behavior, 44, 117–120. 10.1016/j.yebeh.2015.01.004 25678032

[brb33056-bib-0025] Van Den Munckhof, B. , Gefferie, S. R. , Van Noort, S. A. M. , Van Teeseling, H. C. , Schijvens, M. P. , Smit, W. , Teunissen, N. W. , Plate, J. D. J. , Huiskamp, G. J. M. , Leijten, F. S. S. , Braun, K. P. J. , Jansen, F. E. , & Bölsterli, B. K. (2020). Sleep slow‐wave homeostasis and cognitive functioning in children with electrical status epilepticus in sleep. Sleep, 43(11), 10.1093/sleep/zsaa088 32374855

[brb33056-bib-0026] Veggiotti, P. , Termine, C. , Granocchio, E. , Bova, S. , Papalia, G. , & Lanzi, G. (2002). Long‐term neuropsychological follow‐up and nosological considerations in five patients with continuous spikes and waves during slow sleep. Epileptic Disorders, 4(4), 243–249. 12600810

[brb33056-bib-0027] Vinayan, K. P. , Biji, V. , & Thomas, S. V. (2005). Educational problems with underlying neuropsychological impairment are common in children with Benign Epilepsy of Childhood with Centrotemporal Spikes (SeLECTSS). Seizure: The Journal of the British Epilepsy Association, 14(3), 207–212. 10.1016/j.seizure.2005.01.009 15797356

[brb33056-bib-0028] Wu, L. , Yang, X. , Zhang, K. , Wang, X. , & Yang, B. (2021). Impairment of eye emotion discrimination in benign childhood epilepsy with centrotemporal spikes: A neuropsychological study. Brain and Behavior, 11(6), e2154. 10.1002/brb3.2154 PMC8213938 33942564

[brb33056-bib-0029] Xiaoting, L. (2018). Changes of emotional memory in patients with thalamic infarction. Anhui Medical University.

[brb33056-bib-0030] Yan, S. , & Xiaoming, L. (2018). Study on cognitive function of children with benign epilepsy accompanied by spikes in the central temporal region. Chinese Journal of Reproductive Health, 29(03), 297–300.

